# Natural Compounds That Modulate the Development of the Fungus *Botrytis cinerea* and Protect *Solanum lycopersicum*

**DOI:** 10.3390/plants8050111

**Published:** 2019-04-26

**Authors:** Esteban D. Rosero-Hernández, Javier Moraga, Isidro G. Collado, Fernando Echeverri

**Affiliations:** 1 Química Orgánica de Productos Naturales, Instituto de Química, Universidad de Antioquia, Cl. 62 #52–59, Medellín, Colombia; fernando.echeverri@udea.edu.co; 2 Departamento de Química Orgánica, Facultad de Ciencias, Universidad de Cádiz, 11510 Puerto Real, Cádiz, Spain; javier.moraga@uca.es (J.M.); isidro.gonzalez@uca.es (I.G.C.)

**Keywords:** *Botrytis cinerea*, gray mold disease, tomato, control, non-biocide, natural products, 3-phenylpropanol, 1-phenylethanol

## Abstract

*Botrytis cinerea* is the causal agent of gray mold disease and is responsible for the loss of millions of dollars in crops in worldwide. Currently, this pathogen exhibits increasing resistance to conventional fungicides; therefore, better control methods and novel compounds with a more specific mechanism of action but without biocidal effects, are required. In this work, several natural compounds to control *B. cinerea* were analyzed in vitro. Detected effects were dependent on the stage of fungus development, and 3-phenyl-1-propanol displayed the most potent inhibition of in vitro germination, germ tube development, and sporulation. However, it had lower protection of leaves and postharvest fruit in plant infection. Isoeugenol and 1-phenylethanol exhibited lower inhibition of in vitro germination and sporulation, but at the highest concentrations, they inhibited germ tube elongation. Although the lowest rates of foliage infection were recorded using isoeugenol and 3-phenyl-1-propanol, 1-phenylethanol significantly decreased the disease in postharvest tomato fruit, with an efficacy like Mancozeb, but at 18 times lower micromolar concentration. All compounds resulted in high cell viability after spores were removed from the treatment solution exhibited high cell viability, suggesting a non-biocidal effect. The diversity of *in vitro* and in-plant effects seems to indicate a different mechanism of action.

## 1. Introduction

The phytopathogenic fungus *Botrytis cinerea*, the gray mold disease (GMD) agent, is widely distributed throughout the world and infects more than 1400 different species of plants with commercial value both in the field and at the postharvest stage, such as grapes, strawberries, tomatoes, flowers, and ornamental plants, resulting in annual losses of more than 100 billion US dollars globally [1,2]. In tomato greenhouse, the fungus can affect up to 70% of the plants causing a premature death [3].

*B. cinerea* can affect tomato crops by infecting leaves, stems, flowers, and fruits through tissue penetration or wounds, and high amounts of synthetic fungicides are frequently applied. However, the extensive and indiscriminate use of fungicides can cause harmful effects to humans and the environment [4]. Moreover, the biocidal effect generates a selection pressure on the fungus, causing resistance and creating a vicious circle: supplementary fungicides to overcome resistance and therefore higher risk to environment and human health [5]. Due to the economic importance of tomato and the increasing resistance of *B. cinerea* to synthetic fungicides, new alternatives are required to reduce the spread of GMD both in the field and postharvest fruits [6,7]. One crucial strategy implemented for such control has been the use of natural compounds to reduce GMD, such as chitosan, plant extracts, and essential oils [8]. Volatile compounds such as phenylacetic acid, phenyl lactic acid, and phenyl ethanol decrease the growth of some phytopathogenic fungi [9]. However, these natural and synthetic compounds possess a mechanism of action involving a biocidal effect with secondary effects in the environment and on human health.

Thus, this work aimed to determine the in vitro non-biocidal activity of some natural compounds on the growth and development of *B. cinerea*, and subsequently to evaluate the effect on tomato plant leaves and postharvest fruits. This study would allow prospective molecules against GMD, whether pure or mixed with conventional fungicides, to improve their activity and decrease the resistance and virulence.

## 2. Results

From an initial screening of 52 compounds, the ones that were most active against the radial growth of *B. cinerea* on agar plates (results not shown) were selected and were then analyzed in a wide range of concentrations on other growth parameters (Table 1). The assayed compounds displayed high variability of biological activity depending on compound concentration and stage of fungal development. Some of them showed strong effects on the germination and elongation of germ tubes, whereas others acted on a more advanced stage of growth, such as sporulation. All these effects are summarized in Table 2 and are discussed in detail below.

### 2.1. Effects of Compounds on Several Growth Parameters

In the search for molecules with antifungal action, the effect on the germination and development of the hypha is usually determined. However, it is essential to establish a wide range of activities, both in vitro and in plant.

#### 2.1.1. Conidia Germination

**Figure 1:**
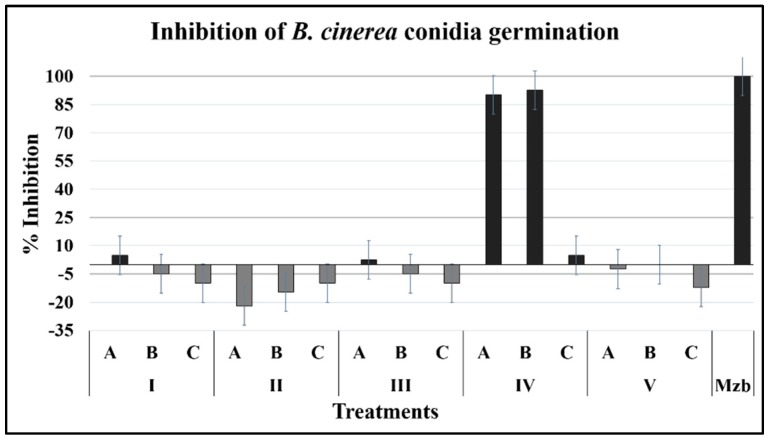
Effect of compounds on conidia germination of *B. cinerea*. Germination was evaluated 12 h after inoculation of medium containing the specified compound (24 °C, 100 rpm).

The germination was evaluated from conidia recovered from a 12-day culture and subjected to treatments during 12 h of incubation. The compound 3-phenyl-1-propanol at 1000 and 500 ppm (IVA and IVB, equivalent to A = 146.8 and B = 73.4 µM, respectively) showed the most potent effect on the germination of *B. cinerea* after 24 h (Figure 1), with an inhibition like that of the control, Mancozeb (2957.4 μM), which completely inhibited the germination of the conidia. The other compounds did not cause effects; some even promoted the germination of conidia by up to 20%, as in the case of the compound IIA (Table 1).

**Figure 2:**
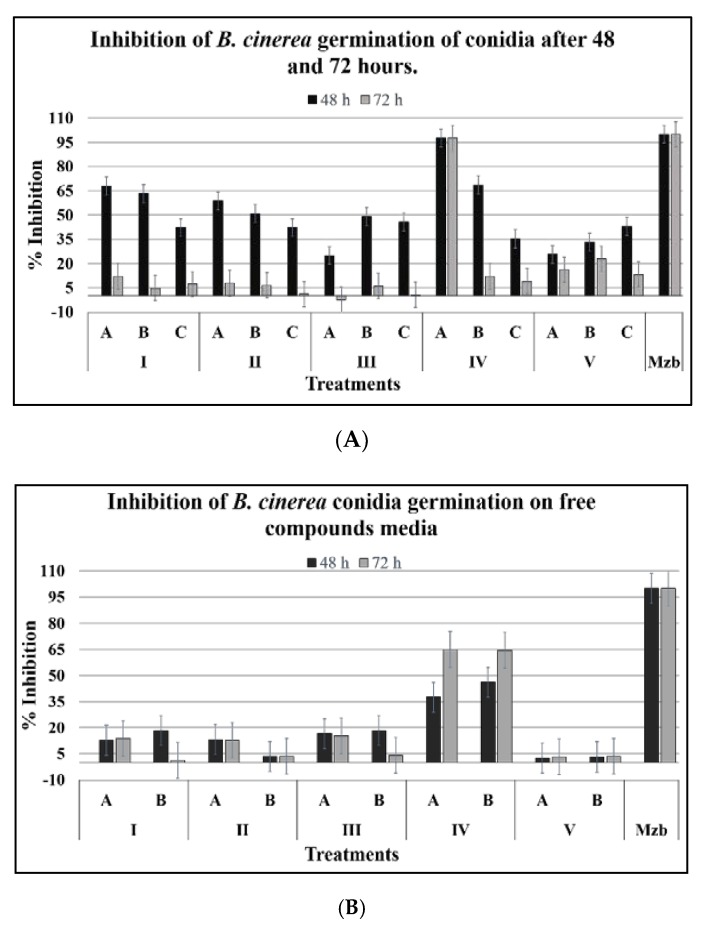
Effect of compounds on *B. cinerea* conidia viability. The inhibition of germination in conidia exposed to treatments (**A**), and in conidia washed and inoculated on agar free of compounds (**B**) was evaluated. The abbreviation referred to structures shown in Table 1.

The conidia were incubated for 48 and 72 h more to determine the long-term effects of the compounds. At the end of this period, the inhibitory effect was significant with the compound 3-phenyl-1-propanol (IVA) with an inhibition value closer to 100% at 48 and 72 h (Figure 2A). After this, the conidia were recovered and grown in fresh media; inhibition of germination of 37.5% and 65.0% were noticed after 48 and 72 h of exposure, respectively, thus demonstrating a non-biocidal effect. The remainder of the compounds presented an inhibition of under 20.0% after 72 h (Figure 2B).

#### 2.1.2. Development of Germ Tubes

The length of the germinative tubes of *B. cinerea* was measured through digital image analysis to compare the effect of compounds on the process of germination of conidia.

**Figure 3:**
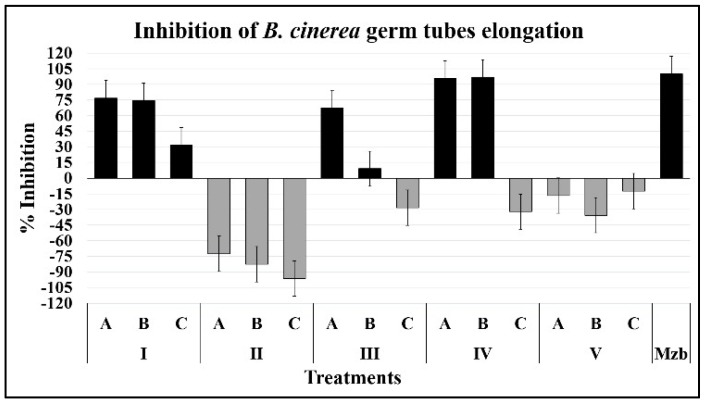
Inhibition of germ-tube growth of *B. cinerea*. The conidia were pregerminated (2 h) and then exposed to the compounds for 12 h. Germ-tube lengths were estimated manually using ImageJ software.

The compound 3-phenyl-1-propanol (IV) had a similar effect to the chemical control, inhibiting the germ-tube development significantly at 1000 and 500 ppm (A = 146.8 and B = 73.4 µM, respectively) (Figure 3). Additionally, isoeugenol (IA = 121.8 and IB = 60.9 µM) and 1-phenylethanol (IIIA = 163.7 µM) also inhibited the development, by almost 80%. Likewise, a marked germination-promoting activity was exhibited with the application of 2-(3-hydroxyphenyl)-ethanol (II), which almost doubled the germ-tube length (IIC = 14.5 µM).

#### 2.1.3. Sporulation

**Figure 4:**
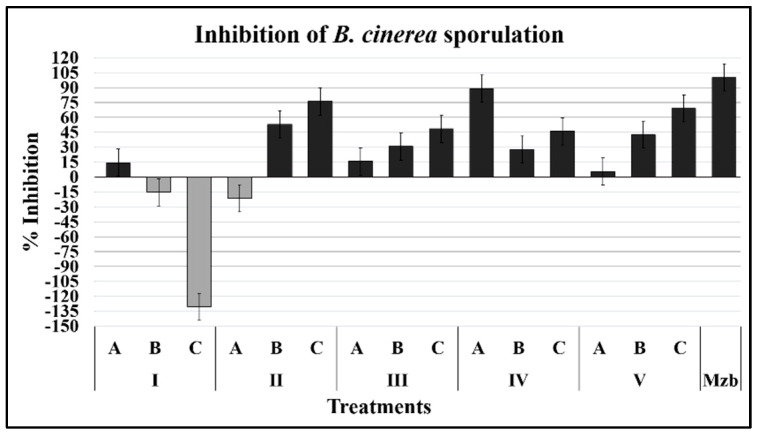
Sporulation inhibition of *B. cinerea*. Conidia were recovered from cultures after 15 days (24 °C, photoperiod 12 h) of incubation and exposed to the test compound for 12 h.

The sporulation of *B. cinerea* was determined in 15-day cultures, growing on Sabouraud agar and inoculated from conidia pretreated with the compounds for 12 h and using culture medium without compounds as controls. All compounds except isoeugenol affected *B. cinerea* sporulation (Figure 4); once again, the compound 3-phenyl-1-propanol at the highest concentration (IVA = 146.8 µM) displayed the most significant inhibitory effect, while 2-(3-hydroxyphenyl)-ethanol (II), 1-phenylethanol (III), and 2′-hydroxyphenylacetic acid (V) showed a high inhibitory effect at lower concentrations. Surprisingly, isoeugenol at the lowest concentration (IC = 12.9 µM) was a strong promoter of sporulation.

### 2.2. Phytopathological Tests

The incidence and severity of *B. cinerea* infection in detached leaves and tomato postharvest fruits exposed to conidia were estimated, as described below.

#### 2.2.1. Effects on Foliage Infected with *B. cinerea*

**Figure 5:**
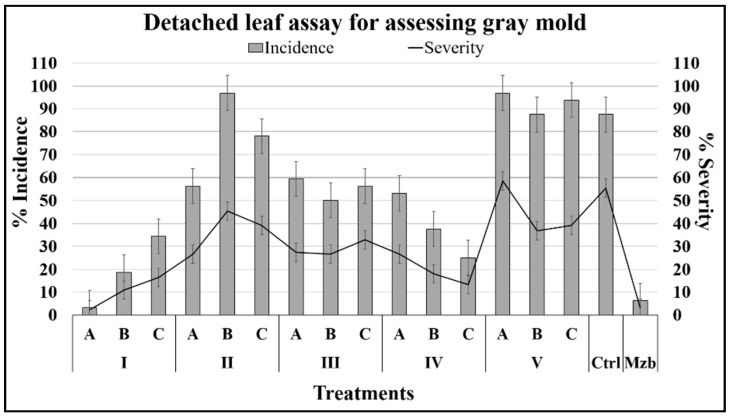
Incidence and severity of *B. cinerea* infection in tomato leaves detached and treated with compounds. Incidence is denoted by the gray bars and severity by the solid black line.

At seventh day of incubation, Isoeugenol (IA = 121.8 µM) strongly reduced the symptoms of GMD in tomato detached leaves, and showed the lowest incidence and severity values, which were similar to those of Mancozeb (Figure 5). In contrast, although isoeugenol decreased the plant infection, weak inhibition of in vitro germination was observed in this work. On the other hand, the compound 3-phenyl-1-propanol, at the lowest concentration (IV = 14.7 µM), also decreased the incidence and severity of GMD, although to a minor degree. This behavior may be a similar response to the bacterial communication *Quorum Sensing*, in which self-inducing molecules modulate pathogenicity and virulence at lower concentrations [10].

#### 2.2.2. Effects on Fruits Infected with *B. cinerea*

**Figure 6:**
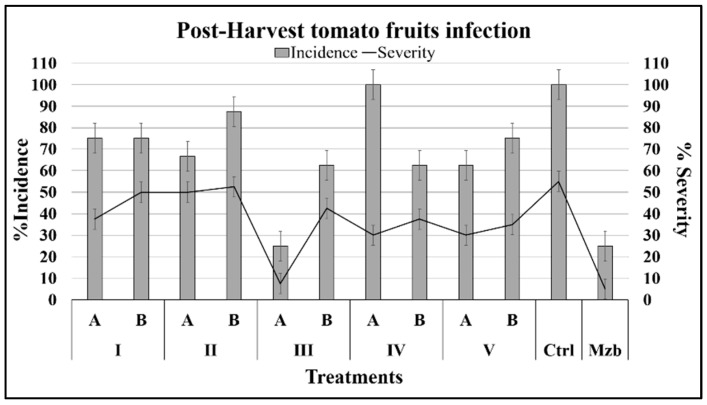
Incidence and severity of the disease caused by *B. cinerea* in postharvest tomato fruits treated with various compounds. The incidence is denoted by the gray bars and the severity by the solid black line.

The tomato postharvest fruits were inoculated with conidia pretreated with the compounds by a superficial wound on the peduncle, and finally at the fifth day the incidence and severity of the infection were determined. In fruits, the incidence and severity of infection were like those of the pathogenic control assay at concentrations of 100 and 50 ppm of compounds (*p* ˃ 0.05 versus control). Nevertheless, at higher concentrations, 1-phenylethanol (IIIA = 163.7 µM) significantly inhibited the development of infection, and the level of protection reached was similar to that of the chemical control Mancozeb (*p* < 0.05) (Figure 6). The 3-phenyl-1-propanol (IVA = 146.8 µM) did not affect the incidence of infection compared with the pathogenic control (*p* ˃ 0.05) although it decreased the severity by 45%. In the presence of isoeugenol and 2-(3-hydroxyphenyl)-ethanol, the severity was highest among the assayed compounds and closest to that of the control group.

## 3. Discussion

The inhibitory activity of 3-phenyl-1-propanol (IVA = 146.8 µM and IVB= 73.4 µM) against conidial germination and germ-tube development is very relevant for its potential use in the field. Thus, treatment could diminish plant diseases caused by the conidial propagule of *Botrytis* by disrupting the early events of infection, such as germination, adhesion to the surface of the host, and the formation of infection structures [11]. Due to its low toxicity and antibiotic properties, this substance (IV) has been used industrially for cosmetic production, mainly as an adjuvant and preservative [12]; therefore, it is a particularly promising compound for crop protection.

Similarly, isoeugenol inhibited germ-tube development by close to 80%, depending on concentration. It is known that some phenolic compounds such as isoeugenol have dual activity, inhibiting the growth of phytopathogen fungi and inactivating metabolizing enzymes, thus obviating the need for detoxification of antifungal compounds [13].

Concerning sporulation, it was determined that early exposure to 3-phenyl-1-propanol (IVA = 146.8 µM) significantly inhibits subsequent sporulation. The inhibitory capacity of this compound has also been reported in *Neurospora crassa* and *Phytophthora cactorum* [14,15].

In contrast to the inhibitory effect on germ-tube development, isoeugenol (IC = 12.9 µM) enhanced the sporulation of *B. cinerea* by almost 125%. One of the main routes of *B. cinerea* infection is the conidia, and although the viability of these structures is reduced, high amounts of conidia from small areas of the mycelium are produced, so it is essential to reduce the propagule in the field.

The compounds isoeugenol, 3-phenyl-1-propanol, and 1-phenylethanol strongly reduced symptoms of disease in tomato leaves, although the most exciting effect was obtained with the last one. The protection to foliage was inversely proportional to the concentration used, with the most significant inhibition of infection being observed at the lowest concentration, suggesting a different mechanism to that of conventional fungicides. Kfoury (2016) evaluated the activity of several phenylpropanoids, on *B. cinerea*, and identified isoeugenol as a potent inhibitor of conidial germination [16]. However, our results showed a low activity of isoeugenol for this parameter, which could be linked to a low susceptibility of the strain to the compound.

Some aromatic alcohols, such as phenylethanol, are known to be produced by some filamentous fungi and yeast acting as *Quorum Sensing* molecules [17]. However, at high concentrations, they can cause toxicity to the fungus and possibly to the host [18]. In *Aspergillus flavus*, a high concentration of 2-phenylethanol completely inhibited fungal growth (fungicide effect), whereas a low concentration promoted growth and inhibited the production of aflatoxins [19]. Besides, this compound has been described in *B. cinerea* and is a part of a type of natural products known as microbial volatile organic compounds (MVOCs) [20], a group of microbial antagonistic compounds proposed as an alternative to fungicides by affecting the population dynamics [21].

As observed, the effect of 1-phenylethanol at low concentrations decreased the gray mold on tomato leaves compared with the chemical control, although without a biocidal effect. Thus, the modulation of pathogenicity is involved, suggesting a concentration-dependent effect as in the *Quorum Sensing* mechanism [22]. In uredospores of wheat rust, the self-inhibition of germination by the production of isomers of methyl-ferulate occurs in response to the high cell density; the exogenous application of the compound in a contaminated crop could decrease the infective capacity of fungus, like our results [23].

Although the compound 3-phenyl-1-propanol at the highest concentrations powerfully inhibited the in vitro development of *B. cinerea* and reduced infection on detached leaves, it showed a low protective effect on tomato fruit. This compound has been reported previously on tomato fruits but at lower concentrations [21]. In turn, the compound 1-phenylethanol protected both tomato foliage and fruit against *B. cinerea* infection. Thus, this abundant and inexpensive natural compound could be a promising candidate for further development and field application to control *B. cinerea*.

On the other hand, the microbial virulence is characterized by high cellular proliferation, with the production of toxins and the formation of a biofilm. If levels of microbial population are maintained, low cell damage would be less critical. The exposure of pathogens to biocide substances quickly creates a selective survival pressure generating the appearance of mutants and resistance. Non-biocide compounds only affect biochemical process without compromising the life process, and the successive application of these type of compounds could be able to control the pathogen. Therefore, it is essential to determine cell viability in avoiding a biocide concentration.

The mechanism of action of these molecules on fungi is unknown, although isoeugenol, 2-(3-hydroxyphenyl)-ethanol and 2′-hydroxyphenylacetic acid could act through an antioxidative effect. However, some results appear to be incompatible since low concentrations of 1-phenylethanol and 3- phenyl-1-propanol inhibit germ tube elongation, but high concentrations promote it (Figure 3); we can explain these observations with two approaches. Firstly, sometimes low concentrations of fungicides cause changes in fungi, such as increases in growth and severity in the disease. This process has been called hormesis and is a risk to crops if it is not appropriately handled [24]. Secondly, although hormesis is recognized in a wide range of cells, seems to be similar to *Quorum Sensing* in bacteria, because at low concentration cell proliferation and other metabolic processes are activated, as has been demonstrated with penicillin [25,26], but a high concentration this compound is an antibiotic. We consider that some of the assayed substance are acting through a *Quorum Sensing* mechanism; however, even though it has been determined, this process is not widely demonstrated in filamentous fungi, and only has been studied in a few fungi such as *Aspergillus* and *Penicillium*. So, we need more evidence like studies on changes in morphology, biofilm formation, and phytotoxin production, among others, to establish the mechanism of action of these compounds.

## 4. Materials and Methods

### 4.1. Equipment and Software

Growth parameters were measured by digital image analysis. Images were obtained using a conventional digital camera (16 MP, f/1.8, 28 mm, LG Electronics, Seoul, South Korea) and a conventional optical microscope (Nikon Alphaphot-2 YS2, Nikon Imaging Japan Inc., Shinagawa Intercity Tower C, 2-15-3, Konan, Minato-ku, Tokyo, Japan).

The determination of germ-tube length was conducted using ImageJ [27]. The data were stored in Microsoft Excel 2016 Office Suite and processed using Statgraphics Centurion XVI (Statgraphics Technologies, Inc., The Plains, VA, USA).

### 4.2. Fungal Strain

*B. cinerea* was isolated from infected tomato fruits (Chonto variety) with the characteristic symptoms of GMD. The disease appears as grey-brown mass of spores covering the infected tissue and in addition to superficial spots with pale- or pale-yellow halo at the beginning [28]. Fruits were purchased in a local market (Medellín, Colombia). A small portion of the sporulated aerial mycelium was extracted, and serial dilutions were made to obtain a highly diluted propagule; from this conidia suspension, monosporic cultures were established on a potato dextrose agar (PDA) medium. The strain was taxonomically related to the *Botrytis* genus and was further identified at the molecular level by the (National Center of Genomic Sequencing; Universidad de Antioquia, Colombia) through amplification of molecular markers ITS1 and ITS4 and the genes *HSP60* and *RPB2*.

### 4.3. Plant Material

Tomato seedlings (*S. lycopersicum* cv Chonto) with 3–5 true leaves were obtained from a commercial producer (Impulsemillas S.A.S, Bogotá, Colombia) and cultivated under controlled pathogen-free laboratory conditions (photoperiod 12 h, dark/light 21/23 °C). The fully extended true leaves were separated from the base of the stem and arranged in Petri dishes lined with sterile moistened filter paper.

Chonto tomato fruits were obtained from an organic farm (Municipio de la Ceja, Antioquia, Colombia). They were selected by size, maturity (mature-green 2–3), and absence of mechanical injury, insect damage, or diseases. Before use, they were washed and disinfected (2% NaClO, 3 min).

### 4.4. Treatments

From an initial screening of 52 natural compounds (Sigma–Aldrich, St. Louis, MO, USA), five were selected to evaluate fungal growth (Table 1). The initial selection criteria were that none of the selected compounds had biocidal effect (80% of viability at 96 h), the phenotypical changes, and the promotion or inhibition of the radial growth of *B. cinerea* with respect to the control. Also, the protective effects of these compounds were evaluated against infection of leaves and postharvest fruits.

The concentrations were established in ppm (mg/L) to determine linear correlations among different activities and assayed at 100, 500, and 1000 ppm, hereafter expressed as A, B, and C, respectively; equivalent values in µM are supplied in Table 1. In tables and figures, the roman numbers I–V represent the compounds evaluated, and the letters A–C the specified concentrations.

All substances were prepared in a mixture of sterile distilled water (SDW) and TWEEN 20 (0.05%). Sabouraud broth (30 g/L) (Sigma–Aldrich, St. Louis, MO, USA) + sterile distilled water and TWEEN 20 (0.05%) was used as the vehicle control and Mancozeb was the chemical control. Mancozeb was prepared at 2 g/L as recommended on the product label (Manzate 200 WP, Dupont Wilmington, DE, USA).

### 4.5. Evaluation of Fungal Growth Parameters

#### 4.5.1. Effect on Germination

Conidia were recovered from the fungus according to a method modified from Slawecki (2002). Briefly, 20 mL of SDW was added to fungus culture growing on Sabouraud agar (24 °C/photoperiod 12 h). The culture surface was then scraped gently with a glass handle to remove the conidia from mycelium. The conidial suspension was filtered and diluted to a final volume of 2 mL (1 × 10^5^ conidia mL^−1^) in Sabouraud broth plus test compound at the indicated concentration [29]. The conidia suspensions were incubated for 12 h (24 °C) under darkness with constant agitation (100 rpm); germination was determined in a Newbauer chamber. Positive germination was indicated when the length of the germ tube was higher than that exhibited by the conidia. Each treatment was evaluated in triplicate, each formed of 50 observations.

The long-term exposure on the germination and the non-biocidal effect of the compounds were established by conidia treatment to compounds and incubation for 48 and 72 h. Then, conidia were washed three times with SDW and incubated once again on Sabouraud agar without any compound for 48 and 72 h to establish the cell viability.

#### 4.5.2. Effect on Germ Tube Formation

Ten milliliters of Sabouraud broth in a 100 mL Erlenmeyer flask was inoculated with conidia recovered from 12-day-old cultures and incubated to induce germination (1 × 10^5^ conidia mL^−1^, 100 rpm, 24 °C). After two hours of incubation, the selected compound was added. The conidia were incubated for 12 h under the conditions mentioned above and germ-tube length was determined by digital image analysis. The treatments were evaluated in triplicate with 20 observations in each case.

#### 4.5.3. Effect on Sporulation

A method modified from Pinedo et al. was used to determine the effect of each compound on fungal sporulation [30]. Ten μL of each culture incubated for 12 h as described in Section 4.5.2 was inoculated in the center of a Petri dish with Sabouraud agar and incubated for 15 days (24 °C, photoperiod 12 h) before recovery and counting of conidia. The conidia were recovered as described in Section 4.5.1 and counted in a Newbauer chamber.

### 4.6. Phytopathological Assays

#### 4.6.1. Protection of Detached Leaves

**Figure 7:**
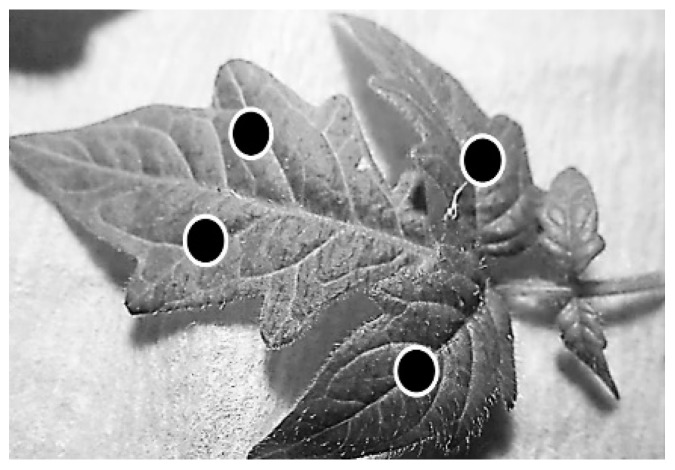
Inoculation of detached *Solanum lycopersicum* leaves with 10 μL of *B. cinerea* conidia suspension (1 × 10^5^ conidia mL^−1^) in four sites (i.e., black circles).

Detached true tomato leaves (four-to-six weeks old) were inoculated at four points with 10 μL of conidia suspension (1 × 10^5^ conidia mL^−1^) pretreated with a test substance as Section 4.5.1 (Figure 7).

Each treatment was estimated at 32 inoculation points distributed over eight leaves. Inoculated leaves were incubated for seven days at 24 °C under a 12 h photoperiod. The incidence and severity of infection were evaluated by Equations (1) (equation of incidence of infection) and (2) (equation of severity of infection) modified from Mouekouba [31].

% I = ((#Inocula with positive infection)/(# Total inoculations)) × 100(1)

% S = Σ(nxv)/VN × 100,(2)

where n = number of positive infections; v = severity level; V = highest level of the severity scale; and N = total number of inocula.

To determine the severity of the infection, a four-level scale was defined according to phenological status: 0 = total absence of infection; 1 = appearance of brown lesions, chlorosis, and/or formation of microsclerotia; 2 = nascent necrotic and chlorotic lesions on the leaf and appearance of aerial mycelium; and 3 = necrosis of the leaf and appearance of sporulated mycelium.

#### 4.6.2. Protection of Tomato Fruit

Tomato fruits were inoculated in the peduncle area through the superficial wound (3 mm long × 2 mm deep) with 50 μL of conidia treated as described in Section 4.5.1. The fruits were incubated for five days (relative humidity = 90–100%, 12 h light, 22–24 °C) until phytopathological evaluation. Incidence and severity were calculated using Equations (1) and (2), respectively; five levels were established to estimate the severity: 0 = absence of infection; 1 = 0–25% infected; 2 = 25–50% infected; 3 = 50–75% infected; and 4 = 75–100% infected.

### 4.7. Statistical Analysis

A completely random design was used. All parameters were subjected to analysis of variance (ANOVA) and a post-hoc analysis for multiple comparisons least significant difference (LSD) test (*p*-value = 0.05), using the Statgraphics Centurion XVI software (Statgraphics Technologies, The Plains, VA, USA). The data that did not fulfill statistical assumptions were analyzed by the comparison of means.

## 5. Conclusions

The effects of several natural compounds on different phenological stages of *B. cinerea* were studied. These were dependent of the concentration and the phenological state, with some compounds showing greater bioactivity during the early stages of development, such as germination, while others were preferentially influencing in more advanced stages, such as sporulation.

The compounds 3-phenyl-1-propanol and 1-phenylethanol showed the ability to control gray mold. The first strongly inhibited early phases of the infective process, including conidia germination, elongation of germ tubes, and sporulation, while the second inhibited fruit disease development; in both cases, a non-biocidal effect was evidenced. At lower concentration, the compound 3-phenyl-1-propanol reduced foliage disease at lower concentration but was not effective in fruit disease control at any concentration, evidencing a preventive role. According to our observations and the information reported by other authors, the activity of 1-phenylethanol during tomato pathogenesis is similar to a *Quorum Sensing* mechanism acting in a concentration-dependent manner. Nevertheless, this assertion should be confirmed, and it is necessary to elucidate the genes involved in this phenomenon.

The potential efficacy of the other natural substances investigated in this study should not be ruled out. For example, isoeugenol significantly decreased the incidence and severity of foliage infection, although fruit protection was weak. Therefore, compound mixtures including isoeugenol may achieve broad protective effects.

Finally, mixtures of some natural compounds like those reported and fungicides could be used to overcome the current microbial resistance, to produce more effective activity, or to reduce the dosage and application frequency.

## Figures and Tables

**Table 1:** Selected substances for biological tests.

Compound	Name *	Micromolar Equivalents		Molecular Structure
		[ppm]	[µM]	
Isoeugenol	**I**	A (1000)	121.8	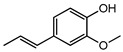
		B (500)	60.9	
		C (100)	12.9	
		D (50)	6.09	
2-(3-hydroxyphenyl)-ethanol	**II**	A (1000)	144.8	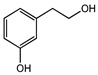
		B (500)	72.4	
		C (100)	14.5	
		D (50)	7.24	
1-Phenylethanol	**III**	A (1000)	163.7	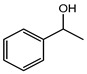
		B (500)	81.8	
		C (100)	16.4	
		D (50)	8.2	
3-Phenyl-1-propanol	**IV**	A (1000)	146.8	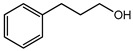
		B (500)	73.4	
		C (100)	14.7	
		D (50)	7.34	
2´-Hydroxyphenylacetic acid	**V**	A (1000)	131.4	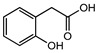
		B (500)	65.7	
		C (100)	13.14	
		D (50)	6.6	
Mancozeb (chemical control)	**Mzb**	2957.4 ^†^		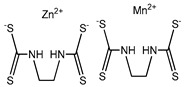

* In the text, compounds are described by a roman number and a letter. ^†^ Micromolar concentration according to the recommended amount on the label (Dupont^®^).

**Table 2:** General effects of various compounds on *B. cinerea* growth parameters.

Treatments and Nomenclature	Parameters Evaluated; Percentage of Inhibition (%)			Tomato Assays			
	Germination	Germ Tubes	Sporulation	% Incidence; Leaves	% Severity; Leaves	% Incidence; Fruits	% Severity; Fruits
Isoeugenol 1000 ppm (IA)	19.5	76.7	14.5	3.1	2.3	75	37.5
Isoeugenol 500 ppm (IB)	14.6	74.3	−15.4	18.8	10.9	75	50
Isoeugenol 100 ppm (IC)	4.9	31.5	−130.4	34.4	16.4	100	55
Isoeugenol 50 ppm (ID)	24.4	−50.7	−85	43.8	23.4	100	54.8
2-(3-Hydroxyphenyl) ethanol) 1000 ppm (IIA)	43.9	−72.5	−21.4	56.3	25.5	66.7	50
2-(3-Hydroxyphenyl) ethanol) 500 ppm (IIB)	7.3	−82.7	53	96.9	45.3	87.5	52.5
2-(3-HYdroxyphenyl) ethanol) 100 ppm (IIC)	29.3	−96.3	76	78.1	39.1	100	56
2-(3-Hydroxyphenyl) ethanol) 50 ppm (IID)	26.8	−60.9	30.6	87.5	35.2	100	55.3
1-Phenylethanol 1000 ppm (IIIA)	31.7	67.4	15.7	59.4	27.3	25	7.5
1-Phenylethanol 500 ppm (IIIB)	19.5	9	30.7	50	26.6	62.5	42.5
1-Phenylethanol 100 ppm (IIIC)	12.2	−28.5	48.4	56.3	32.8	100	55.6
1-Phenylethanol 50 ppm (IIID)	−4.9	−15.4	48	21.9	8.6	100	54.3
3-Phenyl-1-propanol 1000 ppm (IVA)	87.8	95.7	88.9	53.1	25.6	100	30
3-Phenyl-1-propanol 500 ppm (IVB)	80.5	96.5	27.8	37.5	18	62.5	37.5
3-Phenyl-1-propanol 100 ppm (IVC)	17.1	−32.2	46.1	25	13.3	100	52.5
3-Phenyl-1-propanol 50 ppm (IVD)	14.6	−27.9	68.3	84.4	46.1	100	55.3
2-Hydroxy phenyl acetic acid 1000 ppm (VA)	56.1	−16.9	5.6	96.9	53.6	62.5	30
2-Hydroxy phenyl acetic acid 500 ppm (VB)	41.5	−35.6	42.5	87.5	36.7	75	35
2-Hydroxy phenyl acetic acid 100 ppm (VC)	22	−12.6	69	93.8	39.1	100	54.5
2-Hydroxy phenyl acetic acid 50 ppm (VD)	19.5	−12.4	54.6	90.6	32	100	52.5
Control	-	-	-	87.5	55.5	100	55
Mancozeb	100	-	-	6.3	3.1	25	5

Negative values are inversed effects: promotion of germination, tube germ growth and sporulation.
